# Diagnostic dilemma: application of real-time PCR assays for the detection of *Dientamoeba fragilis* in medical and veterinary specimens

**DOI:** 10.1186/s13071-025-06730-1

**Published:** 2025-03-07

**Authors:** Luke M. Hall, John T. Ellis, Damien J. Stark

**Affiliations:** 1 School of Life Sciences, University of Technology Sydney, Broadway, NSW 2007 Australia; 2 Division of Microbiology, Sydpath, St Vincent’s Hospital, Darlinghurst, NSW 2010 Australia

**Keywords:** *Dientamoeba fragilis*, Zoonosis, Molecular detection, Diagnostics

## Abstract

**Background:**

Real-time PCR (qPCR) diagnostics developed for use in human clinical settings have been implemented to identify new animal hosts of the gastrointestinal protozoan *Dientamoeba fragilis*. The gut microbiome varies between species; unrecognised cross-reactivity could occur when applying these assays to new animal hosts. The use of qPCR diagnostics was assessed for the identification of new animal hosts of the gastrointestinal protozoan *Dientamoeba fragilis*.

**Methods:**

Forty-nine cattle, 84 dogs, 39 cats and 254 humans were screened for *D. fragilis* using two qPCR assays: EasyScreen (Genetic Signatures) and a laboratory-based assay commonly used in Europe. The reliability of the identifications made by these assays were assessed using melt curve analysis of qPCR products, conventional PCR targeting the SSU rDNA sequencing and NGS amplicon sequencing of qPCR product.

**Results:**

PCR products from the *D. fragilis* identified in cattle had a 9 °C cooler melt curve than when detected in humans. This melt curve discrepancy, indicative of cross-reactivity with an unknown organism, was investigated further. DNA sequencing determined that *Simplicimonas* sp. was the genera responsible for this cross-reactivity in cattle specimens. *Dientamoeba fragilis* was not detected in either dogs or cats. There was a discrepancy in the number of positive samples detected using the two qPCR assays when applied to human samples. The EasyScreen assay detected 24 positive samples; the laboratory-based assay detected an additional 34 positive samples. Of the discrepant samples, 5 returned sequence data for *D. fragilis*, and 29 were unsupported (false) positive samples.

**Conclusions:**

Analysis of the melt curve after the qPCR reaction is a valuable technique to help differentiate samples containing *D. fragilis* compared to cross-reactions with non-target organisms. The identification of new animal hosts requires further evidence from either microscopy or DNA sequencing to confirm the presence of *D. fragilis*. Additionally, to reduce the risk of false-positive results due to non-specific amplification, we recommend reducing the number of PCR cycles to less than 40. Based on these results, we consider the ramifications of this identified cross-reactivity to the known host species distribution of *D. fragilis*.

**Graphical Abstract:**

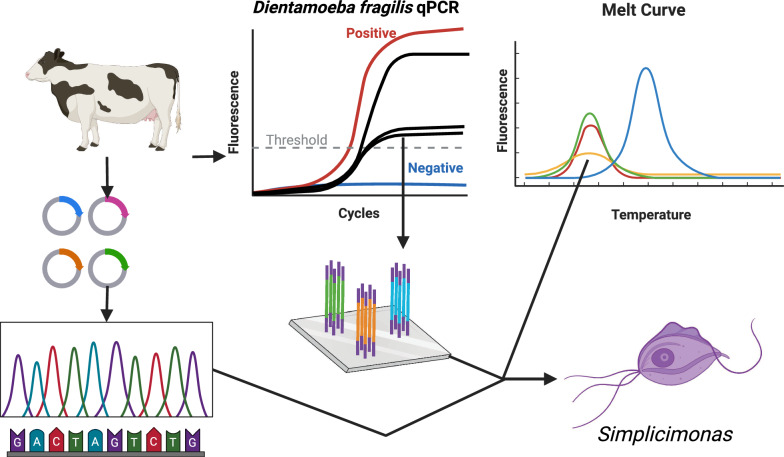

**Supplementary Information:**

The online version contains supplementary material available at 10.1186/s13071-025-06730-1.

## Background

*Dientamoeba fragilis* is a gastrointestinal protozoan parasite initially described in 1918 [1]. After its original description as a commensal [1], this idea was refuted when *D. fragilis* was described as the cause of gastroenteritis in a US military officer [2]. While there is still no consensus on the clinical significance of *D. fragilis*, multiple studies have shown correlation between infection and gastrointestinal symptoms [3, 4]. In the century since the discovery of *D. fragilis*, competing theories have been developed regarding its transmission between hosts. There are two dominant theories: via a helminth vector, identified as *Enterobius vermicularis*, because the closest relative of *D. fragilis*, *Histomonas meleagridis*, is known to use a similar strategy [5]. The second is the faecal-oral transmission of a cyst stage, which was produced in a rodent system [6] but is rarely observed in human clinical samples [7]. *Dientamoeba fragilis* is therefore an organism still shrouded in mystery, and many research questions remain to be answered from a public health perspective.

The One Health perspective recognises that people’s health is inextricably linked to the health of animals and our environment. This perspective has particular significance in the field of parasitology as many parasites are known to have multiple hosts or life cycle stages, allowing the use of a variety of strategies to complete their life cycles. Without a thorough understanding of these relationships, it is impossible to provide control measures and eradication strategies based on expert knowledge. This was exemplified in the recent difficulties of the Guinea Worm Eradication Program after it was discovered that Guinea worms cas use dogs as an alternative host to humans [8]. This example alone highlights the importance of understanding the breadth of hosts when investigating control strategies and the epidemiology of parasites.

From a One Health perspective, there has been a recent trend in research on *D. fragilis* to investigate potential new hosts beyond the non-human primates in which infections were described by microscopy [9, 10]. Several additional hosts have been reported using a variety of molecular techniques including conventional PCR (cPCR) assays combined with DNA sequencing [11–13] and real-time PCR diagnostic assays (qPCR) developed for the screening of human clinical specimens [14–17]. Potential hosts identified include rats [16], cattle [11], budgerigars [17], pigs [12, 18], cats and dogs [14, 15], rabbits, horses, goats and guinea pigs [14]. It is important to note that, other than being detected in non-human primates, these results have not been confirmed through additional studies. For example, two studies from Italy detected *D. fragilis* in pigs with a high prevalence (≈46%) [12, 18]. When 156 pigs from Australia and 116 from Denmark were analysed, *D. fragilis* was not detected [15, 19], raising the possibility that different animals may act as hosts for *D. fragilis* in different regions. While two studies have reported *D. fragilis* in cats and dogs, neither included microscopy or sequence data, with these identifications made solely based on qPCR data [14, 15]. With the recent increase in potential animal hosts identified, it is foreseeable that more animal hosts will be discovered.

Evidence on human infection with *D. fragilis* varies greatly depending on geographical location, study design and methodology. Even when focusing on studies using qPCR techniques, reports on its prevalence vary significantly from 2% [20] up to 71% [21] depending on the cohort being investigated. *Dientamoeba fragilis* often shows a marked age distribution, with young children and their primary caregivers at the greatest risk of infection [21]. Multiple studies have indicated that travel, particularly international, is another significant risk factor [4, 14, 22, 23], including migration [24, 25]. The identification of several hosts of *D. fragilis* raises the possibility that zoonotic transmission plays a role in how humans acquire a *D. fragilis* infection. There is currently one example in the literature where the same genotype of *D. fragilis* was found in infected pigs and their handlers through analyses of a section of SSU rDNA [12]. Supporting this concept, Jirků et al. [14] noted that *D. fragilis* infection was linked to contact with farm animals, while no link was found with contact with pets. Another study investigating the risk factors in childcare centres noted no increased risk associated with pets at home [23]. While there is still a lack of significant amounts of data and additional studies, these findings may indicate that farm animals, rather than pets, may be a zoonotic source of infection. Of the two leading theories on the transmission of *D. fragilis,* the faecal-oral transmission of the cyst stage is more compatible with zoonosis than the *E. vermicularis* vector. *Enterobius vermicularis* is a human-specific helminth with rare identifications in other primates [26]. As such, investigating the zoonotic transmission potential of *D. fragilis* could provide critical insights into how humans acquire an infection and which transmission model is correct/predominant.

In this article, we investigate the use of two real-time PCR assays, initially developed for identifying *D. fragilis* in humans, and look at their effectiveness when applied to DNA from faecal specimens of animals. The microflora of human stool varies significantly from those of other animals where *D. fragilis* has been identified, which provides a possibility for these assays to cross-react with species they have previously been untested against. This potential was shown when the EasyScreen^™^ assay was tested against cultured trichomonads and found to cross-react with *Pentatrichomonas hominis*, which was discriminated from *D. fragilis* through melt curve analysis [15]. Although this knowledge has been in the public domain for over 5 years, recent studies on *D. fragilis* have failed to confirm the identification of those organisms found in hosts such as cattle, dogs and cats. Additionally, in this study we will compare the reliability of these diagnostic assays in a human pathology setting.

## Methods

### Sample collection

All animal samples collected were split into two portions. The first was added to a SAF fixative collection tube (Thermo Fisher) to preserve cell morphology for microscopy analysis. The second was left free of preservatives for use in molecular investigations. A total of 49 samples were collected from cattle. These samples came from animals originating from multiple properties in the Greater Sydney area and were collected from either the cattle property or sale yards. Additionally, 84 samples were collected from dogs and 39 from cats across three council-run animal care facilities. All samples collected were obtained from the ground of pens/cages for individually housed dogs and cats during routine cleaning; animal ethics approval was not required for this process.

To compare the two qPCR assays in a pathology setting, 254 de-identified human clinical samples submitted to a pathology laboratory over an 11-day period were used. Samples were deidentified by removing all patient information and replacing it with an alpha-numeric code to identify samples (A-001 to A-254). No information regarding the origin of these specimens was collected. Human ethics approval was received from St Vincents Hospital Sydney (2021/ETH00961) for using de-identified clinical samples for culturing and molecular research of *D. fragilis*.

### Real-time PCR assay: EasyScreen^™^ assay

The samples were analysed using the EasyScreen Enteric Protozoan Detection Kit (Genetic Signatures) per the manufacturer's instructions. Extraction preparation included transferring faecal material using a sterile swab into a conversion reagent until a colour change was observed. This multiplex PCR assay includes an extraction control and an internal positive control, which detects PCR inhibition and determines whether the sample has been successfully extracted. In cases where samples were found to be inhibited, they were diluted one to five using the kit's initial reagent and subsequently retested. Following the manufacturer's amplification protocol, an additional melt curve analysis was completed by ramping the temperature from 40 °C to 80 °C in 1 °C graduations. The expected melt curve value for *D. fragilis* using the EasyScreen assay is 63 °C to 64 °C [15]. The melt curve values were compared between the human true-positives and animal positive samples using an unpaired two-way t-test with Welch’s correction in GraphPad Prism version 10.1.0.

### Real-time PCR assay: laboratory-based protocol

DNA for a laboratory-based qPCR protocol was extracted using the QIAamp Fast DNA Stool Mini Kit (Qiagen) in conjunction with qPCR Extraction Control Kit (Meridian Bioscience). DNA extraction from 200 mg faecal material followed the manufacturer’s recommendations for the Isolation of DNA for Pathogen Detection method with three modifications: (i) the stool suspension in InhibitEX buffer was heated for 10 min; (ii) 5 µl Internal Control DNA from the qPCR Extraction control kits was added per reaction along with buffer AL; (iii) the incubation time after adding the elution buffer to the spin column was increased to 5 min.

All samples underwent *Dientamoeba fragilis* qPCR using a laboratory-designed assay that amplified a 98-bp fragment within the 5.8 rRNA gene sequence, which will be referred to as the laboratory-based qPCR [27]. The amplification was carried out using the forward primer sequence Df-124F (5′—CAACGGATGTCTTGGCTCTTTA—3′) and reverse primer sequence Df-221R (5′—TGCATTCAAAGATCGAACTTATCAC—3′). In addition, a *D. fragilis*-specific MGB TaqMan probe (Df172revT) was used with the following sequence: 6-carboxyfluorescein (FAM)-5′—CAATTCTAGCCGCTTAT-3′MGB (Thermo Fisher). Each reaction was conducted in a 25-µl volume of PCR buffer (HotstarTaq master mix; Qiagen, Germany), 5 mM MgCl2, 1.5 pmol of each *D. fragilis*-specific primer, 2.5 pmol of *D. fragilis*-specific MGB probe, 2 µl of the DNA sample and 1 µl of the Control Mix containing the primers and probe for the extraction control. Amplification was performed at 95 °C for 15 min followed by 50 cycles of 15 s at 95 °C, 30 s at 60 °C and 30 s at 72 °C. The *D. fragilis* qPCR was performed on the Mic Real-Time PCR System (Bio Molecular Systems).

### Microscopy

Saline was added to fixed specimens before centrifugation at 500 RCF after which the supernatant was removed and discarded. The pellet was then used to make thick and thin films that underwent a modified iron haematoxylin staining procedure. The modifications included adding a carbol fusion stain to visualise coccidia and extending the 50% pitirc acid decolourisation to 4 min to increase differentiation of amoeba from faecal debris. Detailed observation of the stained slides using the 40 × and 100 × objectives of a light microscope was completed to look for protozoa and check for any cells resembling any stages of *D. fragilis*.

### Conventional PCR and cloning

DNA extracted for the laboratory-based qPCR was used for conventional PCR (cPCR). Ten false-positive cattle samples and two human *D. fragilis*-positive samples were amplified and cloned to characterise the species closely related to *D. fragilis* present in the samples. The primer pair, DF400 (5′-TATCGGAGGTGGTAATGACC-3′) and DF1250 (5′-CATCTTCCTCCTGCTTAGACG-3′), targets the SSU rDNA and amplifies an ≈ 850 bp product [28]. PCR reactions were set up using PuReTaq Ready-To-Go (RTG) PCR Beads (Cytiva) in 25 µl reaction volume containing 1 µM of each primer and 1.5 µl of template DNA. After an initial denaturation step at 95 °C for 10 min, 40 cycles were completed: denaturation at 95 °C, annealing at 64 °C and extension at 72 °C, each for 1 min. The final extension step was extended to 10 min to facilitate cloning. Target bands were purified using Size Select II (2%) E-gels (Invitrogen) and stored at − 20 °C if cloning was not directly performed.

The cPCR bands for the primer pair DF400/DF1250 were cloned using a TOPO TA Cloning Kit (Invitrogen). Plasmid DNA was extracted from transformants using the Isolate II plasmid mini kit (Bioline). The target product was amplified using the sequencing primers M13R and M13F (− 20) included in the cloning kit. Cloned amplificons at the appropriate size (≈1000 bp) were purified using the Size Select II (2%) E-gels and Sanger sequenced in both directions by the Australian Genome Research Facility (AGRF).

### Bioinformatics

Sequence data from AGRF were analysed using Geneious Prime (Version 2024.0.5) to generate a consensus sequence from both the forward and reverse sequencing reads. Consensus sequences were trimmed to remove DF400 and DF1250 primers. Trimmed reads were BLAST searched against the NCBI nucleotide database to identify matches. Unique consensus sequences and exemplar sequences from the NCBI Nucleotide database were aligned in MEGA 11 (version 11.0.13) using the muscle alignment tool.

Exemplar sequences were for the target organism, *D. fragilis* (AY730405), and other closely related *Parabasalia* species, ensuring all species identified in the BLAST analysis were: *Histomonas meleagridis (AF293056)*, *Parahistomonas wenrichi (EU647889)*, *Pentatrichomonas hominis (*AF124609), *Trichomonas vaginalis (*TVU17510) and *Tritrichomonas foetus* Tritrichomonas augusta (AY055802), *Tritrichomonas suis* (AY055800)*, Tritrichomonas mobilensis* (AY055801), *Simplicimonas* sp. ( KJ101559, KC953859, GQ254637 and GQ254638), *Monocercomonas colubrorum* (DQ174303), *Tricercomitus* sp. (PP297451), *Trichomonas tenax* (TTU37711), *Trichomitus batrachorum* (MH321568), *Hypotrichomonas blattarum* (KJ591552), *Hypotrichomonas mariae* (HQ149966), *Pimpavicka limacoides* (OK584309) and Undescribed Parabasalid (AB183887).

The best Maximum Likelihood model for building phylogenetic trees for these alignments was determined by using MEGA X to find the best DNA model for each alignment based on having the lowest calculated Bayesian information criterion. Phylogenetic trees were then generated using bootstrapping 500 times using the GTR + G + I model with five discrete gamma categories. *Pimpavicka limacoides* was set as the outgroup and the tree was displayed as a cladogram.

### Next-generation sequencing

Amplicon sequencing of the product generated by the laboratory-based real-time protocol was completed to investigate the identity of the organisms detected in this assay. Genomic DNA used for the initial qPCR assay was sent to AGRF for Illumina MiSeq nano. Custom amplification using Nextera primers (Df-124F and Df-221R) was used to generate the product for sequencing. These primers are the same primers used for qPCR. To mimic the qPCR assay, 50 cycles were completed to generate amplicons for sequencing. Sequence data were then processed in Genious Prime (version: 2024.0.5). Primer sequences were removed from the ends of all sequences which were trimmed using BBDuk with a minimum quality score of 30 and minimum length of 40 bp. Forward and reverse reads were merged with BBMerge using the highest merge rate. Merged sequences between 45 and 55 bp were extracted and OTUs were identified by de novo assembly using the Geneious assembler using the custom settings: maximum gap size 1, allowing for a maximum of 2% mismatches per read and a word length of 10. These OTUs were searched by BLAST against the NCBI nucleotide database to identify their closest match. Representative *Parabasalia* species were included in the custom sequence classification database in addition to those previously detected in this study, those closely related to *D. fragilis* or those commonly found in these human or cattle hosts. The custom database included *D. fragilis* (DQ233458), *Simplicimonas similis* (GQ254635), *Hypotrichomonas acosta* (AY349192), *Tritrichomonas foetus* (MK250822), *Pentatrichomonas hominis* (PHU86616) and *Histomonas meleagridis* (HM229780). Sequences were then classified against the custom database using the Geneious Classify Sequences plugin requiring a minimum overlap of 45 bp and 90% overlap identity to classify. The raw MiSeq data are uploaded to the NCBI Bioproject database under the ID number PRJNA1217120: accession numbers SAMN46479268 to SAMN46479312.

### Comparison of qPCR assays

The 254 human clinical samples were processed using the same workflow as the animal samples for qPCR analysis. Samples in which both qPCR assays detected *D. fragilis* and the GS melt temperature was 63–64 °C or had sequence data aligning with *D. fragilis* were classified as true positives. Samples in which only one assay returned a positive result were submitted for next-generation sequencing using previously mentioned protocol. False-positivity and -negativity rates were used to assess the reliability of both diagnostic assays.

## Results

### Initial qPCR results

**Fig. 1:**
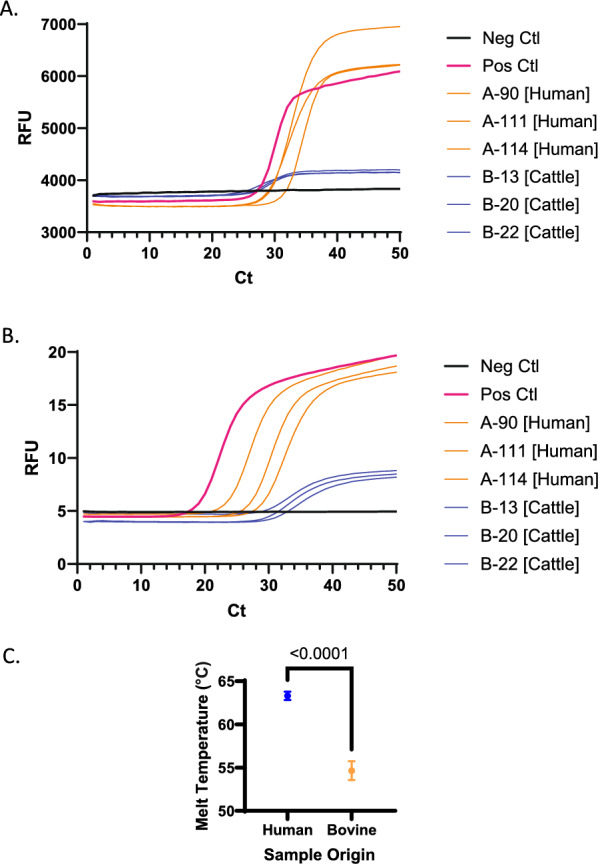
Subset of Real-time PCR curves for EasyScreen assay **A** and laboratory-based assay **B** for the cattle samples in blue and human in orange, plus positive and negative controls. Human and cattle samples have similar Ct values in each assay, respectively, though they differ in fluorescence intensity. Melt curve analysis of EasyScreen Assay **C** shows a significant difference in the ability melting temperature of the probe to anneal when binding to the amplified cattle DNA compared to the human, indicating they are detecting different species

*Dientamoeba fragilis* was not detected in the cat or dog samples included in this study. Initial interpretation of the results from the qPCR assays identified *Dientamoeba fragilis* in 36 out of 49 cattle samples tested using the EasyScreen^™^ and 47 using the laboratory-based protocol. The relative florescence intensity of the quantification curves for both assays was lower for the cattle samples than the human *D. fragilis*-positive samples, indicating a lower amplification/detection efficiency (Fig. 1A and B). Melt curve analysis of the EasyScreen results (Fig. 1c) showed that those positive results in cattle had significantly different melt curves compared to human *D. fragilis* samples, being approximately 9 °C cooler. This difference indicates that the organism detected in the cattle samples was a false-positive result.

### Non-target amplification in cattle samples

No *D. fragilis* was identified in the cattle samples by microscopy. The morphology of protozoa detected in the cattle samples indicates the presence of *Entamoeba* cysts and *Parabaslia* trophozoites (not shown).

**Fig. 2:**
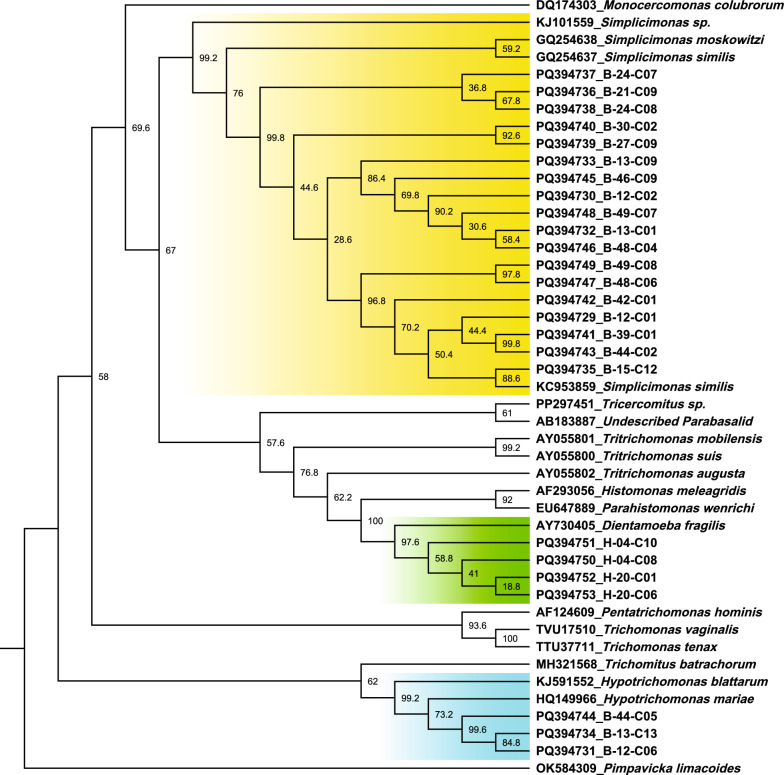
Maximum likelihood phylogeny of DF400/1250 and representative *Parabasalia* species sequence dataset as a cladogram. Nonparametric bootstrap values from 500 bootstrap pseudoreplicates are reported next to the nodes. Tree is rooted on *Pimpavicka limacoides*. Genus clustering with sequence data from clones are highlighted: *Hypotrichomonas* (blue), *Dientamoeba* (green) and *Simplicimonas* (yellow)

For the phylogenetic analysis, a section of the SSU rDNA region was analysed using sequence data from cattle colonised with protozoa cross-reacting with the *D. fragilis* qPCR assays (GenBank accession numbers: PQ394729–49). *Dientamoeba fragilis* sequences from known positive samples were included as controls (GenBank: PQ394750-53). The maximum likelihood tree (GTR + G + I) (Fig. 2) shows the control clade (green) formed by the *D. fragilis* reference sequence and the clones from the human *D. fragilis*-positive samples. Sequencing of other clones from cattle samples with unique sequence reads formed two clusters. The first (yellow) shows clusters with the references sequences for *Simplicionas* sp., the second (green) with *Hypotrichomonas*. Bootstrap values indicate strong support for the clones to cluster with these genera. This identification was further supported in the BLAST analysis.

**Fig. 3:**
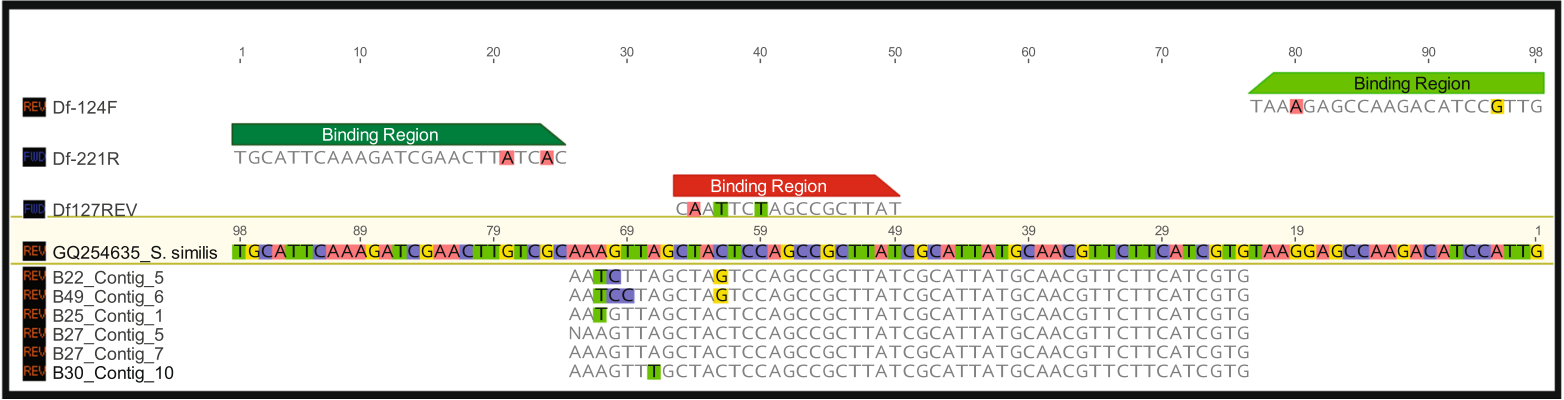
Geneious alignment of NGS amplicon sequences from cattle samples using the laboratory-based assay’s primers. Sequences, primers and probe of the laboratory-based assay are aligned to *Simplicimonas similis* (GQ254635) 5.8S rDNA gene sequence, which was the most similar BLASTn match. There are three nucleotide differences in the 5’ region of the probe (Df127REV)

Illumina MiSeq amplicon data of the laboratory-based assay show that the sequences generated have the closest similarity with the *Simplicimonas* genus, not *Hypotrichomonis* (Fig. 3). There are three nucleotide differences between the probe (Df127REV) and the sequence data generated in this study. Sequence data for the product generated using the commercial EasyScreen assay are unobtainable as it works using 3-base methodology, and the target and primer are commercial secrets.

### Comparison of qPCR

**Fig. 4:**
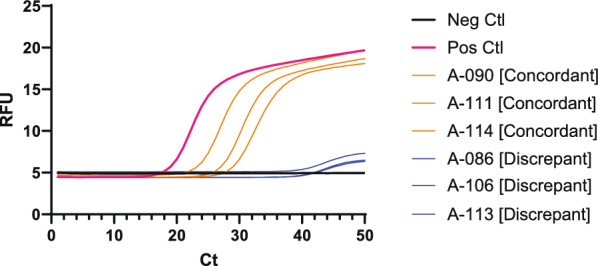
Subset of real-time PCR curves for the laboratory-based *Dientamoeba fragilis* diagnostic assay run on 254 unknown human clinical specimens. Representative samples that were concordant with the EasyScreen Assay are depicted in orange. Discrepant samples with a positive qPCR result in laboratory-based assay but negative in EasyScreen are shown in blue. Amplification for all discrepant samples occurred at approximately Ct 40

There was a large discrepancy between the number of positive results detected using the EasyScreen and the laboratory-based assay on the 254 unknown human clinical samples. Using the EasyScreen assay, 24 samples were positive for *D. fragilis*. These detections were supported by melt curve analysis, which determined the peak dissociation of the probe from the amplified DNA occurred at 63–64 °C, concordant with known samples of *D. fragilis*. All 24 positive samples detected in the EasyScreen assay were also positive using the laboratory-based assay. Using the laboratory-based assay, there were an additional 34 discrepant positives, bringing the total to 58 *D. fragilis*-positive samples detected by the laboratory-based assay. Assessment of the 34 discrepant samples by NGS amplicon sequencing determined that sequence data for *D. fragilis* could be generated in 5 of the 34 discrepant samples. A subset of the amplification curves for these concordant and discrepant positives reveals that detection of the discrepant samples occurs around cycle 40 (Fig. 4). Overall, in this study of 254 human clinical samples, there were 29 true-positive samples (11%), 5 false negative when using the EasyScreen assay, and 29 unsupported (false) positives when using the laboratory-based assay.

## Discussion

When considering the zoonotic potential of *D. fragilis*, it is crucial that we have an accurate understanding of what animal species are possible hosts and determine whether the *D. fragilis* detected in these hosts is the same species as that found in humans. *Dientamoeba fragilis* has previously been reported in all species included in this study. The two studies which previously investigated dogs and cats identified *D. fragilis* in 2–8% of dogs and 1.5–5% of cats in sample sizes < 60 animals [14, 15]. In our study, we analysed 84 dogs and 39 cats without detecting *D. fragilis*. As such, we are unable to provide supporting evidence for dogs and cats as a host of *D. fragilis*, nor can we argue against it, as while we have samples of more dogs and a comparable number of cats compared to both previous studies, our sample size is currently insufficient for us to conclude that *D. fragilis* cannot be present in either host.

Results for the cattle specimens in this study seemed to identify *D. fragilis* in 47 of the 49 samples tested following the developer’s protocols. Following the recommendations of Chan et al. [15], a melt curve analysis on all positive results using the EasyScreen assay determined that the melting temperature for all the positive results in cattle was approximately 9 °C cooler than that of the human *D. fragilis*-positive samples. This difference in melting temperature between human and cattle samples was significant. This difference indicates that the assay is amplifying different DNA in cattle to that found in humans. The scale of this difference is comparable to what was seen when this assay is run against *Pentatrichomonas hominis* [15], indicating that what is being detected in the cattle samples is not a novel subtype of *D. fragilis* but from a different genus entirely. This is supported by sequence data of the SSU rDNA where two genera, *Simplicimonas* and *Hypotrichomonas*, were identified by Sanger sequence instead of *D. fragilis*.

The original determination of cattle as hosts for *D. fragilis* used a conventional PCR assay in combination with sequencing and microscopy [11]. The sequences generated by Yildiz and Erdem Aynur, [11] were similar to those of *D. fragilis* with a similarity 99.75% to the genotype 1 reference sequence. Our sequence data using the same primers identified *Hypotirchomonas* sp. in three samples and *Simplicimonas* sp. in all sequenced samples from cattle (Fig. 2). Cross-reactivity with the laboratory-based assay was confirmed to occur with *Simplicimonas* sp. in the NGS data (Fig. 3). There were three points of mismatch between the probe used in the laboratory-based assay and the *Simplicimonas* sequence data, which is less than the five points of mismatch which has previously been shown to generate a detectable signal with other TaqMan probe base assays [29]. Although we were unable to detect *D. fragilis* in cattle in this study, the previous detection which included sequencing and microscopy still provides strong evidence that cattle are hosts for *D. fragilis* [11].

Although cattle can still be confidently identified as a host of *D. fragilis*, this cross-reactivity has ramifications regarding the recent identification of horses, goats, rabbits and guinea pigs as hosts for *D. fragilis* as no melt curve analysis or sequence data were generated to allow for definitive identification of *D. fragilis* in these animal species [14]. Jirků et al., 2022, were able to generate one DNA sequence from a sample that was shown to contain *D. fragilis* in a rabbit implementing the same laboratory-based qPCR method used in this study. Phylogenetic analysis of this sequence placed it on a separate branch within the clade including *D. fragilis*, *Histomonas meleagridis* and *Parahistomonas wenrichi* when using a SSU rDNA target. In this case, the researcher concluded that this may be a new lineage or genus of a closely related protist.

*Simplicimonas similis* has been described in the literature in a recent reclassification of *Parabasalids* [30]. *Simplicimonas similis* was detected in both poultry and carabao [31, 32]. A study in cattle determined that *Simplicimonas* sp. like DNA was cross-reacting with a qPCR assay for the detection of *Tritrichomonas foetus* in cattle vaginal swabs [33]. Another qPCR, developed to target a region of the SSU rDNA of *D. fragilis* for diagnostic purposes [34], which was not tested in this study, has been shown to cross-react with *S. similis* [35]. The possibility that the two qPCR assays used in this study may also cross-react with the genus *Simplicimonas* raises questions on their reliability and their use in animal specimens for the detection of *D. fragilis*.

Unlike the cattle samples where the false-positive results could be attributed to non-target amplification, most of the discrepant positive results in the human samples could not be attributed to cross-reactivity with a specific organism. Of the 34 samples which were positive in the laboratory-based assay, five were found to have sequence data classified as *D. fragilis* and seven as *Simplicimonas* (two also *D. fragilis* positive). *Simplicimonas similis* was detected in humans with a prevalence of 1.5% in Madagascar [35]. Using only the short 50-bp sequence from the NGS data, we cannot confirm the presence of *Simplicimonas* sp. in these samples. Confirmation using the cloning protocol could not be completed because of insufficient PCR product for cloning and the presence of multiple non-target products when the number of cycles was increased because of a low concentration of target DNA, as indicated by the high Ct values. This is a common issue with late Ct qPCR-positive samples [14]. The remaining 24 samples were unsupported by the sequence data as *D. fragilis* and generated unclassified or low-quality sequence data. This meant that 29 of the 58 positive samples using the laboratory-based assay could not be confirmed by additional evidence. These unsupported (false) positives from the human samples in this study had high Ct values, typically ≥ 40. Higher Ct values are often associated with non-target products, the most likely cause of our result [36]. To limit the risk of false-positive results, future studies using the laboratory-based protocol on human samples are recommended to limit the number of cycles to between 35 and 40. Notably, these false positives were determined by the absence of *D. fragilis* sequence data when these samples were sequenced on the NGS platform. The strength of this evidence is limited as the absence of evidence is not necessarily evidence of absence.

Recent studies investigating *D. fragilis* in humans have often use these qPCR techniques as they are considered the most sensitive [21, 23, 37, 38]. The reported prevalence of *D. fragilis* varies significantly. In pathology samples from Sydney, Australia, the prevalence of *D. fragilis* in humans is 10% using the EasyScreen Assay (unpublished data) or 37.2% using the laboratory-based assay [39]. A similar cohort in Denmark using the laboratory-based assay reported a prevalence of 43% [21]. In our comparison of these two qPCR assays, the laboratory-based assay had a false-positive rate of approximately 50%. If the prevalence in Sydney using the laboratory-based assay is adjusted using this rate, the prevalence is 18.7%, closer in line with the EasyScreen data, which is a slight underestimation (unpublished observations). This false-positivity rate supports the previous study where 31 false-positive reactions with known negative samples were identified [40]. One of the arguments against *D. fragilis* being a pathogen is the high positivity rate in asymptomatic individuals causing *D. fragilis* infection not to correlate with symptoms [23, 41]. Our data indicate that the assay used in these studies has a high false-positive rate, so we suggest this conclusion should be re-examined.

## Conclusions

This study does not provide evidence to support or contradict the presence of *D. fragilis* in cattle; however, the primary finding is that sequence data detected the presence of the related genera *Simplicmonas* and *Hypotrichomonas*. The cross-reactivity of the qPCR assays used to detect *D. fragilis* means that animal hosts cannot be identified solely using this technique by either qPCR assay. Further evidence by melt curve analyses, sequencing and/or microscopy is required for definitive identification. In the future, developing more specific qPCR tests for the detection of *D. fragilis* in animals should be a priority. When researching novel hosts, it is recommended that melt curve analysis be completed in conjunction when using the EasyScreen assay and sequencing of an additional locus when using the laboratory-based protocol to detect *D. fragilis*. When using these assays with human samples, reducing the number of cycles to between 35 and 40 will limit false-positive results, which could lead to patients receiving unnecessary treatments.

## Supplementary Information

Supplementary material 1.

## Data Availability

Sequence data from clone DNA were deposited in Genbank with accession codes PQ394729-PQ394753. Next Generation Sequence data using qPCR primer are deposited in the BioProject PRJNA1217120. Summary of qPCR reactions is provided in the supplementary information.
